# Exploring the causal pathways and mediating effects of sociopsychological factors on chronic coronary syndrome: A Mendelian randomization study

**DOI:** 10.1097/MD.0000000000045971

**Published:** 2025-11-28

**Authors:** Xiaotong Li, Yan Wang, Chunhua Song, Xiaojing Guo

**Affiliations:** a Second School of Clinical Medicine, Heilongjiang University of Chinese Medicine, Harbin, China; b Rehabilitation Center, The Second Affiliated Hospital of Heilongjiang University of Chinese Medicine, Harbin, China; c Department of Acupuncture, The Second Affiliated Hospital of Heilongjiang University of Chinese Medicine, Harbin, China.

**Keywords:** anxiety, chronic coronary syndrome, depression, inflammation, social psychology

## Abstract

This study sought to explore the causal pathways and mediating effects of sociopsychosocial factors on chronic coronary artery syndrome (CCS) from a genetic variation perspective. The Mendelian randomization approach was used to investigate the causal effects of sociopsychological factors (including anxiety, depression, constipation, diarrhea, nausea and vomiting, social deprivation, and family income) on chronic coronary syndrome. For the analysis, the TwoSampleMR, MR-PRESSO, multivariable Mendelian randomization (MVMR), and forestploter packages in R software were employed. The inverse variance weighting method was the primary approach for evaluation of the causal estimates. Anxiety, depression and diarrhea exhibit significant positive causal associations with CCS (*P* *=* .022, *P* = .001, *P* = .000). Household income demonstrated a significant negative causal relationship with CCS (*P* = .014). Furthermore, even after adjusting for anxiety, depression remained significantly and positively associated with CCS (*P* = .028). The mediators implicated in these causal pathways encompass TNF-related apoptosis-inducing ligand (TRAIL), low density lipoprotein (LDL), triglycerides (TG), and smoking initiation. A total of 8 significant mediation pathways were identified involving sociopsychosocial factors, mediator variables, and CCS (proportion of mediating effects): anxiety → smoking initiation → CCS (10.26%), anxiety → TRAIL → CCS (6.67%), anxiety → LDL → CCS (35.86%), major depressive disorder (MDD) → TRAIL → CCS (7.38%), MDD → LDL → CCS (34.20%), family income → smoking initiation → CCS (12.83%), family income → LDL → CCS (20.99%), and family income → TG → CCS (20.64%). Anxiety, depression, and diarrhea were linked to an increased risk of CCS, while higher family income was associated with a lower risk. These factors also showed indirect effects on CCS through smoking behavior, inflammation, and blood lipid levels, offering a theoretical basis for future clinical research.

## 1. Introduction

Chronic coronary syndrome (CCS) has a broad scope, complex pathogenesis, and highly heterogeneous clinical features. Although CCS is a generally stable disease, it can rapidly progress, even leading to acute coronary syndrome, if cardiovascular risk factors are poorly managed (e.g., suboptimal risk control, poor adherence to lifestyle interventions, or ineffective drugs/revascularization).^[1–6]^ Conversely, active management may delay or reverse progression, making CCS patient management a critical clinical priority. Notably, rapid societal changes have spawned persistent social conflicts, driving the accumulation of adverse sociopsychological factors (e.g., stress, anxiety, depression, social deprivation, low family income) that significantly contribute to CCS onset and progression.^[1–3,7]^ Among the psychosomatic diseases linked to such factors, “double-heart disease” is the most prevalent; it is worsening physical symptoms (e.g., chest pain) while reducing treatment adherence and quality of life. Clarifying how these factors affect CCS is key to improving patient outcomes. Despite progress in studying sociopsychological factors and CCS, the field faces major methodological and scientific limitations. Most causal studies remain observational and burdened by unmeasured confounders, hindering efforts to identify core mechanistic links. Animal models, though useful for partly clarifying the biological pathways between sociopsychological stressors and CCS, cannot confirm definitive causality – due to inherent limitations like poor cross-species (e.g., mouse-to-human) translation of pharmaceutical effects and the risk of anthropomorphizing animal behaviors (e.g., equating mouse actions to human anxiety/depression).

Despite notable research efforts, 2 key challenges persist. First, causal evidence is insufficient, and the interactions/combined effects of sociopsychological factors are understudied. Clinically, patients with CCS often face multiple adverse stressors, such as anxiety, depression, economic strain, interpersonal tension, inadequate social support, and sleep disturbances.^[8]^ Observational studies link some emotional states (e.g., anxiety and depression) to CCS incidence, yet the findings are often confounded by factors such as financial hardship, leaving it unclear if factors synergize, counteract, or interact dynamically.^[8,9]^ Second, the underlying mediating mechanisms are poorly understood. The literatures^[10–13]^ suggest that neuroendocrine dysregulation, vascular endothelial dysfunction, and inflammatory activation may mediate the impact of sociopsychological stress on CCS; however, the precise sequence, interplay, and detailed mechanism between these 3 pathways lack clarity or consensus.

Mendelian randomization (MR) is a robust method for addressing the limitations of conventional observational studies. Using genetic variants, particularly single nucleotide polymorphisms (SNPs), as instrumental variables (IVs), MR can reduce confounding factors and reverse causality. Genetic variants are randomly assigned during meiosis, stable over time, and independent of the environment/disease status, strengthening the causal inference between sociopsychological factors (exposures) and CCS (outcome). This study applied MR to explore these causal relationships from a genetic epidemiological perspective, hypothesizing that adverse factors (anxiety, depression, diarrhea, low income) elevate CCS risk, higher income reduces it, and these effects are partially mediated by smoking initiation, TRAIL, LDL, and TG. The aim was to deepen our understanding of psychosocial CCS mechanisms and support targeted prevention and treatment strategies.

## 2. Materials and methods

### 2.1. Research participants

This study utilized publicly available genome-wide association study (GWAS) data. All data used were approved by the relevant institutional review boards of each country in accordance with the Declaration of Helsinki, and all study participants signed informed consent forms. No separate ethical approvals were required for this study.

### 2.2. Data sources

The GWAS data for exposure, outcomes, and mediating factors were downloaded. Table 1 details the data sources.

**Table 1:** Data sources of sociopsychological factors, mediating factors, and chronic coronary syndrome.

Phenotypes	Source	Year	Codes*, references or ICD codes	Sample size	Cases (n)	Controls (n)	Cases (%)
Sociopsychological factors							
Anxiety	GWAS Catalog	2021	GCST90041879	4,44,404	2,51,982	1,92,422	56.7%
MDD	GWAS Catalog	2016	GCST003769	1,05,739	16,471	58,835	15.577%
Constipation	GWAS Catalog	2021	GCST90018829	5,88,252	16,299	5,71,953	2.77%
Diarrhea	GWAS Catalog	2021	GCST90044165	4,56,348	367	4,55,981	0.08%
Nausea and vomiting	UKB	2018	ukb-b-19419	4,63,010	2143	4,60,867	0.463%
Type A behavior	GWAS Catalog	2021	GCST90013454	3268	NA	NA	NA
Social deprivation	GWAS Catalog	2024	GCST90302885	4,40,350	NA	NA	NA
Household income	GWAS Catalog	2019	GCST009523	2,86,301	NA	NA	NA
Mediating factors							
Cytokines	University of Bristol	2020	Laura Corbin, Nicholas Timpson (2020): Cytokines GWAS results. https://doi.org/10.5523/bris.3g3i5smgghp0s2uvm1doflkx9x	8293	NA	NA	NA
LDL	IEU	2013	ieu-a-300	1,73,082	NA	NA	NA
TC	IEU	2013	ieu-a-301	1,87,365	NA	NA	NA
TG	IEU	2013	ieu-a-302	1,77,861	NA	NA	NA
VTE	GWAS Catalog	2022	GCST90399745	10,63,277	27,987	10,35,290	2.632%
Autonomic nervous	GWAS Catalog	2018	GCST90435914	3,95,475	266	3,95,209	0.067%
MVPA	GWAS Catalog	2018	GCST006097	3,77,234	NA	NA	NA
Ever smoking	GWAS Catalog	2019	GCST007327	5,18,633	NA	NA	NA
Smoking cessation	GWAS Catalog	2019	GCST007460	5,47,219	NA	NA	NA
Smoking initiation	GWAS Catalog	2018	GCST90029014	4,68,170	NA	NA	NA
Drink	GSCAN	2019	PMID:30643251	9,41,280	NA	NA	NA
Chronic coronary syndrome							
Coronary atherosclerosis	FinnGen	2024	finngen_R12_I9_CORATHER	88,268	NA	NA	NA

CCS = coronary atherosclerosis, IEU = Institute of Epidemiology and Healthcare, LDL = low density lipoprotein, MDD = major depressive disorder, MVPA = moderate-to-vigorous physical activity, TC = total cholesterol, TG = triglycerides, VTE = venous thromboembolism.

Exposure factors included anxiety, major depressive disorder (MDD), constipation, diarrhea, nausea vomiting, type A behavior, social deprivation, and household income. Mediating factors include cytokines, low density lipoprotein (LDL), total cholesterol (TC), triglycerides (TG), venous thromboembolism, autonomic nervous function, moderate-to-vigorous physical activity (MVPA), smoking cessation, smoking initiation, and drink. Coronary atherosclerosis was the outcome factor. As the concept of CCS was introduced in 2019 and coronary atherosclerotic lesions were more common before 2019, coronary atherosclerosis was chosen as the outcome factor.

### 2.3. Methods

All GWAS summary statistics used in this study were obtained from large-scale studies. For the MR analysis, to ensure samples for exposure and outcome did not come from the same database (to avoid sample overlap effects), the 2 variables were not simultaneously selected from the Finnish or UKB (UK Biobank) databases.

First, a univariate MR analysis was conducted for exposure and outcome using inverse variance weighting (IVW) as the primary method, and combined with MR-Egger, weighted median estimator (WME), simple mode, and weighted mode. After completing these analyses, multivariate MR (MVMR) analysis was performed on exposures that were causally related to the outcome. Two-step MR was then used to conduct an analysis of mediating and outcome factors. Exposures and mediators that were significantly causally related to the outcome (i.e., *P* < .05) were selected, and a second MR analysis was conducted. Finally, the proportion of mediating effect was calculated. The specific methods used are as follows.

#### 2.3.1. Inclusion and exclusion criteria for instrumental variables (IVs)

This study adhered to established principles for selecting IVs for MR analyses. Single nucleotide polymorphisms (SNPs) were chosen based on the following 5 core conditions to ensure the validity and reliability of causal inferences.

1. Strong association with exposures/mediators: SNPs significantly associated with exposures and mediators were extracted from GWAS summary data using the extract_instruments function in the R package “TwoSampleMR.” In general, SNPs meeting genome-wide significance (*P* < 5 × 10⁻⁸) were selected. If too few SNPs met this threshold, the cutoff was relaxed to *P* < 5 × 10⁻⁶ to ensure an adequate sample size and reduce bias from limited SNP availability. SNPs with *P* ≥ 5 × 10⁻⁶ were excluded.
2. Independence among IVs: linkage disequilibrium (LD) clustering analysis was performed to ensure independence among the selected SNPs. For most exposures and mediators, SNPs with an LD parameter *r*² ≤ 0.01 and a physical distance ≥ 10,000 kb were retained. For inflammatory factors, a stricter threshold (*r*² ≤ 0.01 and physical distance ≥ 1000 kb) was applied. SNPs exceeding the respective LD thresholds (i.e., exhibiting strong LD) were excluded to prevent redundancy and potential distortion of the results.
3. No confounding and no horizontal pleiotropy: only SNPs showing no significant association with potential confounders (*P* *>* .05) and influencing the outcome exclusively through exposures or mediators were included.
4. Sufficient strength (avoid weak IVs): the coefficient of determination (*R*²) and *F*-statistic for each SNP were calculated to assess their strength. The *F*-statistic is calculated using the following formula:

$$\begin{array}{l} F=\frac{N-K-1}{K}\times \frac{R^{2}}{1-R^{2}} \end{array}$$

where N is the sample size of the GWAS, *K* is the number of IVs, and *R*^2^ is the proportion of the variance in exposure explained by the instrument. SNPs with an *F*-statistic > 10 were retained to minimize the risk of weak instrumental bias. SNPs with an *F*-statistic ≤ 10 were excluded.

1. Consistent effect size information: the direction and magnitude of effect sizes were harmonized using the “harmonise_data” function in the R package “TwoSampleMR.” Only SNPs that successfully passed the harmonization process were included. SNPs that failed to align in terms of the effect allele or effect direction (i.e., did not pass effect size harmonization) were excluded.

#### 2.3.2. MR and sensitivity analysis

MR analysis was performed using R software (version 4.3.2; R Foundation for Statistical Computing, Vienna, Austria) and the “TwoSample” package. The IVW method was the primary approach, and the WME, MR-Egger, WME, SM, and weighted mode were used in parallel to assess causal relationships. The results are presented as ORs and 95% CIs. To control the false-positive rate introduced by multiple genetic instruments, the *P*-values of IVW were corrected using the false discovery rate (FDR), with *P* < .05 indicating statistical significance.

To enhance the reliability of the MR analysis results, a sensitivity analysis was conducted that focused on heterogeneity and horizontal pleiotropy analyses. Cochran’s *Q* statistic was used to assess heterogeneity (*P* *>* .05, no heterogeneity). When heterogeneity was present, a random-effects model was used; otherwise, a fixed effects model was used. Horizontal pleiotropy was tested using the MR-Egger intercept method (*P* *>* .05, indicating no significant horizontal pleiotropy). The MR-PRESSO method was used to detect the pleiotropy and identify potential outliers. If *P* *>* .05 occurred in both MR-PRESSO and MR-Egger, there is no horizontal pleiotropy. A funnel plot was used to determine the heterogeneity of the MR analysis results. The leave-one-out method was used to remove individual SNPs to further evaluate the impact of individual SNPs on the stability of the results.

#### 2.3.3. Two-step MR

The first step estimates the causal effect of significant exposure (*X*) on mediator (*M*) (β_*X*→*M*_), and the second step estimates the causal effect of mediator (*M*) on outcome (*Y*) (β_*M*→*Y*_). The indirect effect was β_indirect_ = β_*X*→*M*_ × β_*M*→*Y*_. The total effect was the univariate MR estimate of the significant exposure (*X*) on the outcome (*Y*) (β_total_). Proportion of the mediator effect = β_indirect_/β_total_.

## 3. Results

### 3.1. Univariate MR analysis of social psychological factors and CCS

Following a comprehensive screening of IVs, 156 relevant SNPs were identified across 8 social psychological factor exposures, ranging from 5 to 52 SNPs per exposure. The *F*-statistics for all IVs ranged between 21 and 87, exceeding the threshold of 10 and confirming the absence of weak instruments. Detailed information on the IVs extracted for each sociopsychological factor is provided in Table S1, Supplemental Digital Content, https://links.lww.com/MD/Q674, including SNP identifiers (rs numbers), beta coefficients, standard errors, effect alleles, non-effect alleles, and *P*-values.

Detailed results are presented in Table 2 and Figure 1. In Figures S1–S8, Supplemental Digital Content, https://links.lww.com/MD/Q674 sequentially display the visualization outcomes of the leave-one-out method, funnel plots, and scatter plots.

**Table 2:** Mendelian randomization and sensitivity analysis between sociopsychological factors and chronic coronary syndrome (CCS).

Exposure	Outcome	Method	*P*	OR (95% CI)	Heterogeneity	Horizontal pleiotropy
					IVW_*P*	MR-Eggerintercept_*P*
Anxiety	CCS	IVW	.022*	1.111 (1.015–1.216)	0.001†	0.971
MDD	CCS	IVW	.001*	1.274 (1.097–1.478)	0.965	0.663
Constipation	CCS	IVW	.408	1.022 (0.971–1.075)	0.117	0.693
Diarrhea	CCS	IVW	.000*	1.021 (1.009–1.032)	0.259	0.877
Nausea and vomiting	CCS	IVW	.127	0.000 (1.19E−07 to 7.340)	0.713	0.897
Type A behavior	CCS	IVW	.485	1.107 (0.970–1.066)	0.348	0.966
Social deprivation	CCS	IVW	.458	1.060 (0.908–1.237)	0.238	0.529
Household income	CCS	IVW	.014*	0.651 (0.462–0.918)	0.000†	0.812

CCS = chronic coronary syndrome, CI = confidence interval, IVW = inverse variance weighting, MDD = major depressive disorder, MR = Mendelian randomization, OR = odds ratio.

* A causal relationship in the MR results; IVW_*P* is Cochran’s *Q* value for the heterogeneity test.

† Heterogeneity and a random-effects model is used for analysis.

**Figure 1.**
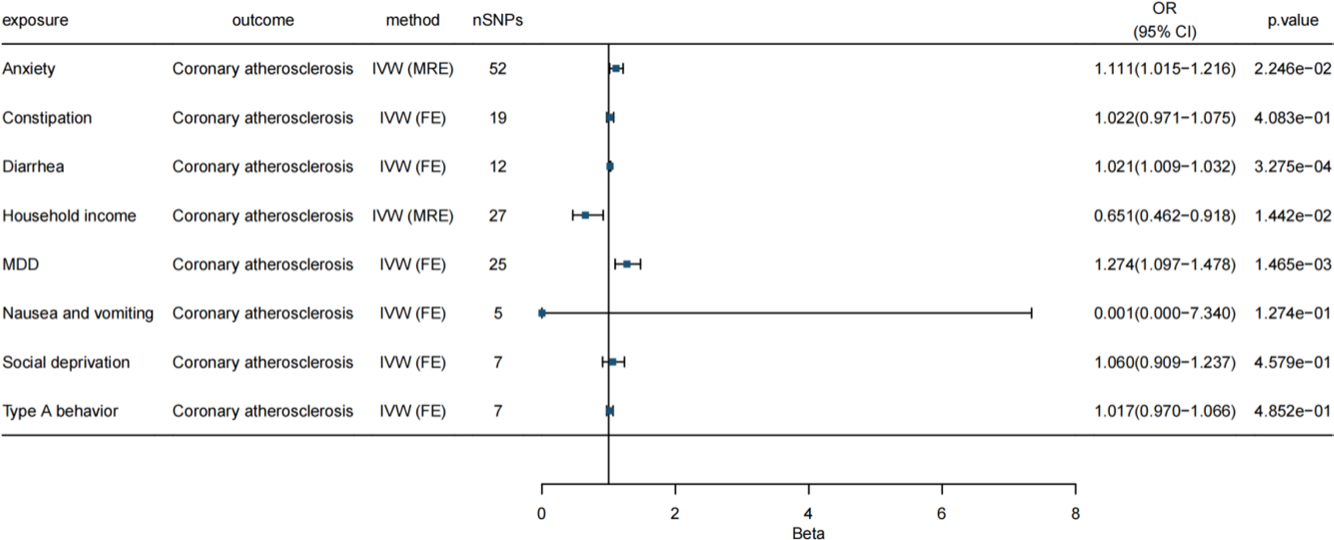
Forest plot of the results of univariate MR analysis of the impact of sociopsychological factors on CCS. IVW (MRE) is the inverse variance weighted method (multivariate random-effects model), and IVW (FE) is the inverse variance weighted method (fixed effects model). An OR > 1 indicated that the exposure factor increased the risk of the outcome, whereas an OR < 1 indicated reduced risk. The 95% CI interval, excluding 1, was statistically significant. Vertical line: 95% confidence interval (95% CI). When it did not cross 1, the association between the exposure factors and coronary atherosclerosis was statistically significant; when it crossed 1, it was not significant. CCS = chronic coronary syndrome, FE = fixed effect, IVW = inverse variance weighting, MDD = major depressive disorder, MR = Mendelian randomization, MRE = multivariate random effects, nSNP = number of single nucleotide polymorphism, OR = odds ratio, SNP = single nucleotide polymorphism.

### 3.2. Multivariable Mendelian randomization (MVMR)

#### 3.2.1. Analysis of significant sociopsychological factors and CCS

**Figure 2.**
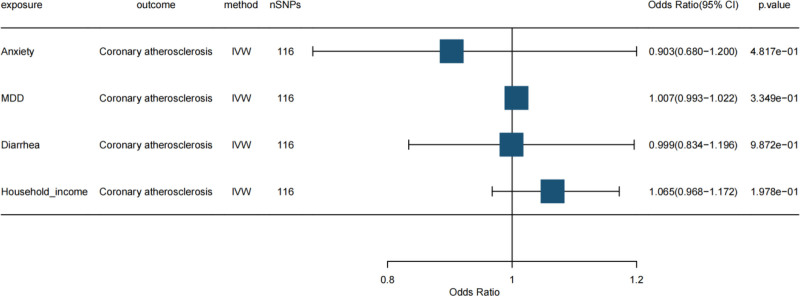
Forest plot of MVMR analysis results. IVW, inverse variance weighted method; nSNPs, number of single nucleotide polymorphisms (single nucleotide polymorphisms). An OR > 1 indicated that the exposure factor increased the risk of the outcome, whereas an OR < 1 indicated reduced risk. The 95% CI interval, excluding 1, was statistically significant. Vertical line: 95% CI. When it did not cross 1, the association between the exposure factors and coronary atherosclerosis was statistically significant; when it crossed 1, it was not significant. CI = confidence interval, IVW = inverse variance weighting, OR = odds ratio, SNP = single nucleotide polymorphism, MVMR = multivariate MR.

In the MVMR analysis examining genetically predicted anxiety and depression as exposures in relation to CCS outcomes, depression demonstrated a significant positive causal association with CCS after adjusting for anxiety (OR: 1.097, 95% CI: 1.010–1.192, *P* = .028). These results are summarized in Tables 3–5 and illustrated in Figure 2.

**Table 3:** Results of multivariable Mendelian randomization analysis of anxiety and major depressive disorder (MDD).

Exposure	Method	*P*	OR (95% CI)	Heterogeneity	Horizontal pleiotropy
				IVW_*P*	MR-Eggerintercept_*P*
Anxiety	IVW	.875	1.013 (0.863–1.188)	0.037†	0.962
MDD	IVW	.028*	1.097 (1.010–1.192)		

CI = confidence interval, IVW = inverse variance weighting, MDD = major depressive disorder, MR = Mendelian randomization, OR = odds ratio.

* A causal relationship.

† Heterogeneity; and a random-effects model was used for analysis.

**Table 4:** Results of multivariable Mendelian randomization analysis of diarrhea and household income.

Exposure	Method	*P*	OR (95% CI)	Heterogeneity	Horizontal pleiotropy
				IVW_*P*	MR-Eggerintercept_*P*
Diarrhea	IVW	.147	1.013 (0.996–1.030)	4.01E−06*	0.439
Household income	IVW	.487	0.881 (0.615–1.261)		

CI = confidence interval, IVW = inverse variance weighting, MR = Mendelian randomization, OR = odds ratio.

* Heterogeneity; and a random-effects model was used for analysis.

**Table 5:** Results of multivariable Mendelian randomization analysis of 4 sociopsycological factors.

Exposure	Method	*P*	OR (95% CI)	Heterogeneity	Horizontal pleiotropy
				IVW_*P*	MR-Eggerintercept_*P*
Anxiety	IVW	.482	0.903 (0.680–1.200)	1.16E−06*	0.415
MDD	IVW	.335	1.007 (0.933–1.022)		
Diarrhea	IVW	.987	0.999 (0.834–1.196)		
Household income	IVW	.198	1.065 (0.968–1.172)		

CI = confidence interval, IVW = inverse variance weighting, MDD = major depressive disorder, MR = Mendelian randomization, OR = odds ratio.

* Heterogeneity; a random-effects model was used for analysis.

### 3.3. Two-step Mendelian randomization analysis

#### 3.3.1. MR and sensitivity analysis between mediators and CCS

Following a comprehensive screening process for IVs, 1022 SNPs were identified, with the number of SNPs per exposure ranging from 4 to 162. The *F*-statistics for all the IVs exceeded 10, confirming the absence of weak instruments.

For detailed results, please refer to Table 6 and Figure 3. Figures S9–S15, Supplemental Digital Content, https://links.lww.com/MD/Q674 sequentially displays the visualization outcomes of the leave-one-out method, funnel plots, and scatter plots.

**Table 6:** Mendelian randomization and sensitivity analysis between mediators and chronic coronary syndrome (CCS).

Mediating	Method	*P*	OR (95% CI)	Heterogeneity	Horizontal pleiotropy
				IVW_*P*	MR-Eggerintercept_*P*
Cytokines					
TRAIL	IVW	.000*	1.033 (1.015–1.051)	0.100	0.537
IL-10	IVW	.000*	0.941 (0.911–0.973)	0.291	0.052
RANTES	IVW	.015*	0.952 (0.915–0.991)	0.671	0.255
CTACK	IVW	.044*	0.974 (0.950–0.999)	0.130	0.658
IL-17	IVW	.000*	0.915 (0.868–0.964)	0.497	0.982
LDL	IVW	1.05E−53*	1.462 (1.393–1.534)	2.879	0.714
TC	IVW	2.68E−30*	1.419 (1.336–1.507)	3.760	0.043†
TG	IVW	1.17E−17*	1.369 (1.274–1.471)	4.189	0.537
VTE	IVW	.012*	1.030 (1.007–1.054)	3.887	0.086
Autonomic nervous	IVW	.443	1.006 (0.991–1.020)	0.618	0.586
MVPA	IVW	.726	1.048 (0.806–1.362)	1.542	0.970
Ever smoking	IVW	.290	1.500 (0.708–3.175)	8.922	0.933
Smoking cessation	IVW	.000*	1.372 (1.157–1.626)	1.993	0.201
Smoking initiation	IVW	.000*	1.221 (1.091–1.367)	5.728	0.030†
Drink	IVW	.741	0.960 (0.751–1.226)	3.572	0.426

IVW_*P* = Cochran’s *Q* value for heterogeneity test.

CI = confidence interval, IVW = inverse variance weighting, LDL = low density lipoprotein, MR = Mendelian randomization, MVPA = moderate-to-vigorous physical activity, OR = odds ratio, TG = triglyceride, TRAIL = TNF-related apoptosis-inducing ligand, VTE = venous thromboembolism.

* A causal relationship in the MR results.

† Presence of pleiotropy.

**Figure 3.**
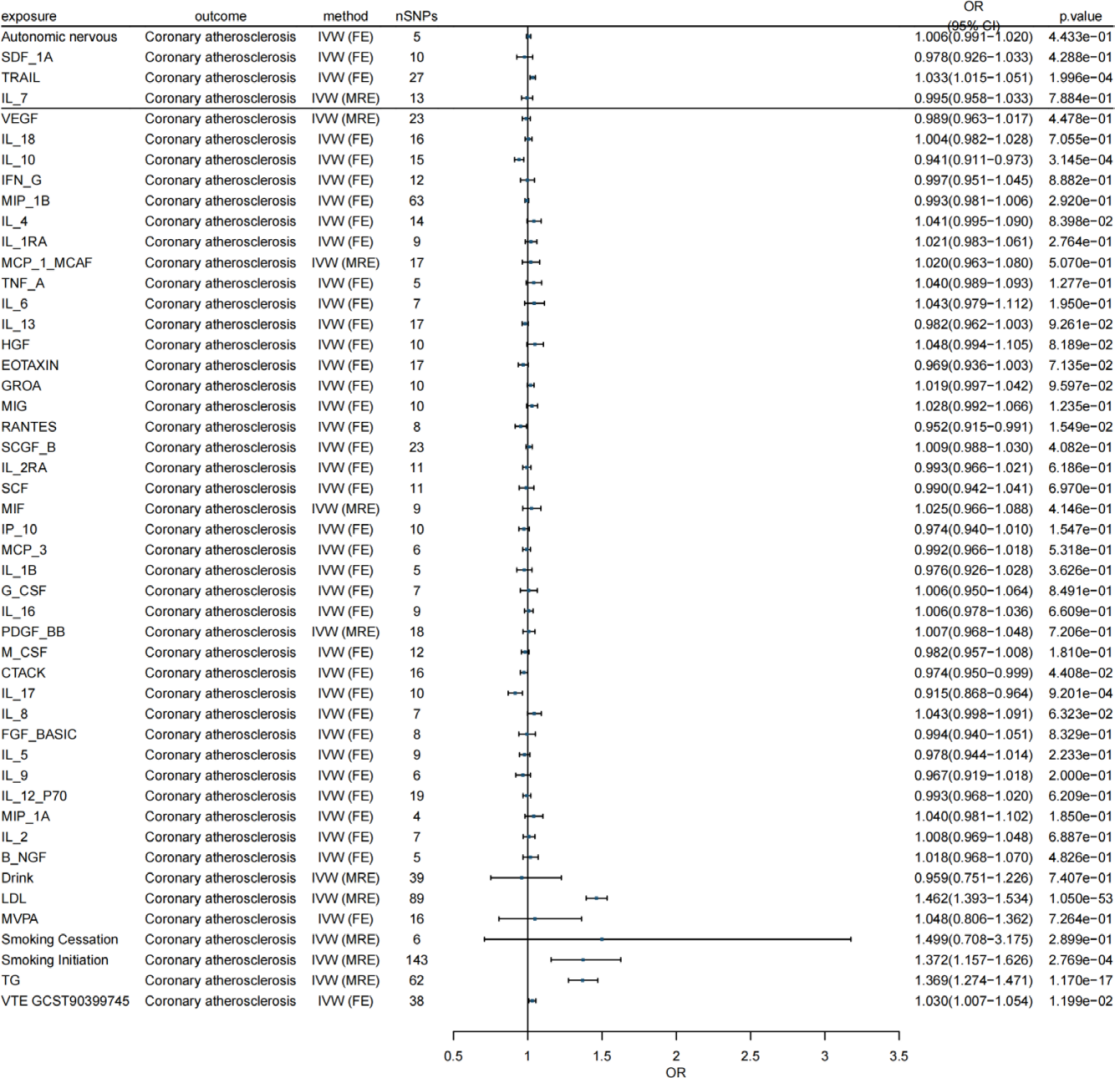
Forest plot of MR analysis results for all mediators, including inflammatory factors. IVW (MRE) is the inverse variance weighted method (multivariate random-effects model), and IVW (FE) is the inverse variance weighted method (fixed effects model). An OR > 1 indicated that the exposure factor increased the risk of the outcome, whereas an OR < 1 indicated reduced risk. The 95% CI interval, excluding 1, was statistically significant. Vertical line: 95% CI. When it did not cross 1, the association between the exposure factors and coronary atherosclerosis was statistically significant; when it crossed 1, it was not significant. CI = confidence interval, FE = fixed effect, CCS = chronic coronary syndrome, IVW = inverse variance weighting MR = Mendelian randomization, MRE = multivariate random effects, OR = odds ratio.

#### 3.3.2. Univariate MR analysis of significant sociopsychological factors and significant mediators

The results from this analysis included 4 aspects:

1. MR analysis of anxiety as an exposure with significant mediators as outcomes. Anxiety demonstrated negative causal associations with TRAIL (OR: 0.805, 95% CI: 0.692–0.937, *P* = .005), VTE (OR: 0.863, 95% CI: 0.763–0.977, *P* = .020), and smoking initiation (OR: 0.966, 95% CI: 0.941–0.992, *P* = .011), with no heterogeneity or horizontal pleiotropy. Furthermore, anxiety showed significant positive causal relationships with RANTES (OR: 1.397, 95% CI: 1.102–1.769, *P* = .006) and LDL (OR: 1.105, 95% CI: 1.026–1.190, *P* = .009), also without heterogeneity or horizontal pleiotropy. For further details, please refer to Table 7.
2. MR analysis of depression as an exposure with significant mediators as outcomes. Depression demonstrated positive causal associations with TRAIL (OR: 1.733, 95% CI: 1.234–2.435, *P* = .002), IL-10 (OR: 1.438, 95% CI: 1.012–2.043, *P* = .043), and smoking initiation (OR: 1.049, 95% CI: 1.004–1.095, *P* = .032), with no heterogeneity or horizontal pleiotropy. Moreover, a significant negative causal relationship was observed between depression and LDL (OR: 0.084, 95% CI: 0.661–0.979, *P* = .030), also without heterogeneity or horizontal pleiotropy. For further details, please refer to Table 8.
3. MR analysis of diarrhea as an exposure with significant mediators as outcomes. There was no suggestive causal association between diarrhea and TRAIL, RANTES, IL-10, CTACK, IL-17, LDL, TGs, VTE, or smoking initiation. For further details, please refer to Table 9.
4. MR analysis of household income as an exposure with significant mediators as outcomes. Household income demonstrated a negative causal association with LDL (OR: 0.789, 95% CI: 0.638–0.976, *P* = .029), TGs (OR: 0.755, 95% CI: 0.619–0.920, *P* = .005), and smoking initiation (OR: 0.840, 95% CI: 0.782–0.903, *P* = 2.35 × 10^−6^), with no heterogeneity or horizontal pleiotropy. For further details, please refer to Table 10.

**Table 7:** Mendelian randomization and sensitivity analysis between anxiety and significant mediators.

Exposure	Mediate	Method	*P*	OR (95% CI)	Heterogeneity	Horizontal pleiotropy
					IVW_*P*	MR-Eggerintercept_*P*
Anxiety	Cytokines					
	TRAIL	IVW	.005*	0.805 (0.692–0.937)	0.887	0.160
	IL-10	IVW	.308	0.922 (0.788–1.078)	0.078	0.702
	RANTES	IVW	.006*	1.397 (1.102–1.769)	0.183	0.817
	CTACK	IVW	.373	0.902 (0.720–1.131)	0.386	0.620
	IL-17	IVW	.453	1.062 (0.908–1.241)	0.418	0.598
	LDL	IVW	.009*	1.105 (1.026–1.190)	0.712	0.874
	TG	IVW	.898	0.994 (0.901–1.096)	0.019†	0.016‡
	VTE	IVW	.020*	0.863 (0.763–0.977)	0.038†	0.710
	Smoking initiation	IVW	.011*	0.966 (0.941–0.992)	5.949	0.815

IVW_P = Cochran’s *Q* value for the heterogeneity test.

CI = confidence interval, IVW = inverse variance weighting, LDL = low density lipoprotein, MR = Mendelian randomization, MVPA = moderate-to-vigorous physical activity, OR = odds ratio, TG = triglyceride, TRAIL = TNF-related apoptosis-inducing ligand, VTE = venous thromboembolism.

* A causal relationship in the MR results.

† Heterogeneity and a random-effects model was used for analysis.

‡ The presence of horizontal pleiotropy.

**Table 8:** Mendelian randomization and sensitivity analysis between depression and significant mediators.

Exposure	Mediate	Method	*P*	OR (95% CI)	Heterogeneity	Horizontal pleiotropy
					IVW_*P*	MR-Eggerintercept_*P*
MDD	Cytokines					
	TRAIL	IVW	.002*	1.733 (1.234–2.435)	0.114	0.658
	IL-10	IVW	.043*	1.438 (1.012–2.043)	0.610	0.413
	RANTES	IVW	.610	0.872 (0.516–1.474)	0.780	0.670
	CTACK	IVW	.598	0.873 (0.526–1.448)	0.216	0.560
	IL-17	IVW	.303	1.202 (0.847–1.706)	0.315	0.781
	LDL	IVW	.030*	0.804 (0.661–0.979)	0.667	0.900
	TG	IVW	.739	1.031 (0.863–1.230)	0.745	0.811
	VTE	IVW	.159	1.165 (0.942–1.441)	0.165	0.817
	Smoking initiation	IVW	.032*	1.049 (1.004–1.095)	2.056	0.288

IVW_*P* = Cochran’s *Q* value for heterogeneity test.

CI = confidence interval, IVW = inverse variance weighting, LDL = low density lipoprotein, MR = Mendelian randomization, MVPA = moderate-to-vigorous physical activity, OR = odds ratio, TG = triglyceride, TRAIL = TNF-related apoptosis-inducing ligand, VTE = venous thromboembolism.

* A causal relationship in the MR results.

**Table 9:** Mendelian randomization and sensitivity analysis between diarrhea and significant mediators.

Exposure	Mediate	Method	*P*	OR (95% CI)	Heterogeneity	Horizontal pleiotropy
					IVW_*P*	MR-Eggerintercept_*P*
Diarrhea	Cytokines					
	TRAIL	IVW	.537	1.010 (0.981–1.038)	0.066	0.304
	IL-10	IVW	.513	1.010 (0.981–1.040)	0.052	0.187
	RANTES	IVW	.949	0.998 (0.952–1.047)	0.065	0.553
	CTACK	IVW	.157	1.032 (0.988–1.079)	0.630	0.921
	IL-17	IVW	.540	1.010 (0.980–1.039)	0.284	0.040*
	LDL	IVW	.993	1.000 (0.981–1.020)	0.084	NA
	TG	IVW	.621	0.996 (0.978–1.013)	0.393	NA
	VTE	IVW	.937	1.001 (0.983–1.019)	0.953	0.554
	Smoking initiation	IVW	.344	1.001 (0.999–1.003)	0.318	0.337

IVW_*P* = Cochran’s *Q* value for heterogeneity test.

CI = confidence interval, IVW = inverse variance weighting, LDL = low density lipoprotein, MR = Mendelian randomization, MVPA = moderate-to-vigorous physical activity, OR = odds ratio, TG = triglyceride, TRAIL = TNF-related apoptosis-inducing ligand, VTE = venous thromboembolism.

* The presence of horizontal pleiotropy.

**Table 10:** Mendelian randomization and sensitivity analysis between household income and significant mediators.

Exposure	Mediate	Method	*P*	OR (95% CI)	Heterogeneity	Horizontal pleiotropy
					IVW_*P*	MR-Eggerintercept_*P*
Household income	Cytokines					
	TRAIL	IVW	.740	1.093 (0.646–1.851)	0.097	0.183
	IL-10	IVW	.938	0.979 (0.568–1.687)	0.538	0.267
	RANTES	IVW	.452	0.731 (0.322–1.656)	0.237	0.813
	CTACK	IVW	.314	0.667 (0.304–1.466)	0.522	0.168
	IL-17	IVW	.892	0.963 (0.560–1.657)	0.704	0.511
	LDL	IVW	.029*	0.789 (0.638–0.976)	0.091	0.403
	TG	IVW	.005*	0.755 (0.619–0.920)	0.485	0.284
	VTE	IVW	.488	0.840 (0.513–1.375)	0.046†	0.646
	Smoking initiation	IVW	2.35E−06*	0.840 (0.782–0.903)	8.441	0.890

IVW_*P* = Cochran’s *Q* value for heterogeneity test.

CI = confidence interval, IVW = inverse variance weighting, LDL = low density lipoprotein, MR = Mendelian randomization, MVPA = moderate-to-vigorous physical activity, OR = odds ratio, TG = triglyceride, TRAIL = TNF-related apoptosis-inducing ligand, VTE = venous thromboembolism.

* A causal relationship in the MR results.

† Heterogeneity and a random-effects model was used for analysis.

#### 3.3.3. Analysis of mediating effects

After the 2-step MR analysis, there were significant mediating effects between the 8 groups of sociopsychological factors and CCS as well as between the mediating variables: anxiety → smoking initiation → CCS, anxiety → TRAIL → CCS, anxiety → LDL → CCS, MDD → TRAIL → CCS, MDD → LDL → CCS, family income → smoking initiation → CCS, family income → LDL → CCS, family income → TG → CCS. The proportions of the 8 mediating effects were 10.26%, 6.67%, 35.86%, 7.38%, 34.20%, 12.83%, 20.99%, and 20.64% respectively (Table 11).

**Table 11:** Mediating effects data table.

Exposure (*X*)	Mediate (*M*)	β_*X*→*M*_	β_*M*→*Y*_	β_total_	β_indirect_	Proportion of mediation effect
Anxiety	Smoking initiation	−0.034	0.316	0.105	−0.011*	10.26%
	RANTES	0.334	−0.049		−0.016	15.62%
	TRAIL	−0.216	0.032		−0.007*	6.67%
	LDL	0.099	0.380		0.038*	35.86%
	VTE	−0.147	0.030		−0.004	4.15%
MDD	Smoking initiation	0.048	0.316	0.242	0.015	6.22%
	IL_10	0.363	−0.061		−0.022	9.08%
	TRAIL	0.550	0.032		0.018*	7.38%
	LDL	−0.218	0.380		−0.083*	34.20%
Household income	Smoking initiation	−0.174	0.316	−0.429	−0.005*	12.83%
	LDL	−0.237	0.780		−0.090*	20.99%
	TG	−0.282	0.314		−0.090*	20.64%

Y = CCS.

CCS = chronic coronary syndrome, CTACK = cutaneous T-cell attracting chemokine; IL-10 = interleukin-10; IL-17 = interleukin-17; LDL = low-density lipoprotein, RANTES = regulated on activation, normal T cell expressed and secreted, TG = triglycerides, TRAIL = TNF-related apoptosis-inducing ligand, VTE = venous thromboembolism.

* There is a significant relationship between exposure to social psychological factors and CCS outcomes through mediator *M*.

## 4. Discussion

The majority of prior studies investigating the causal relationship between psychosocial factors and CCS were observational in nature, with numerous unknown confounding factors. Although animal experiments can elucidate the specific mechanisms of certain social psychological factors in CCS, it remains challenging to verify precise causal effects. MR analysis is an advanced statistical method that leverages genetic variation to assess the causal relationships between sociopsychological factors and CCS. Using SNPs as IVs reduces interference from confounding environmental factors. Therefore, this study explores the causal effects of sociopsychological factors on CCS from a genetic perspective. Multiple GWAS datasets were used, with the IVW method serving as the primary approach for univariate MR analysis. This method was used to examine the causal effects of sociopsychological factors on CCS, their influence on inflammatory factors as mediators, and their effects on CCS. Additionally, a 2-step MR approach was applied to investigate the potential mediating factors and proportion of mediating effects in the causal pathways linking social factors to CCS. This methodology has been widely adopted in previous research.^[14,15]^ Sensitivity analyses were conducted to evaluate the robustness of the findings. The study revealed that anxiety, depression, and diarrhea significantly increased the risk of CCS, whereas higher household income reduced this risk. Notably, the effect of depression on CCS is independent of anxiety; however, both should be considered during the intervention. Anxiety, depression, and household income indirectly influenced CCS through their effects on smoking behavior, inflammatory responses, and blood lipid profiles.

### 4.1. MR analysis of sociopsychological factors on CCS

In this study, anxiety, depression, and diarrhea exerted significant positive causal effects on CCS, whereas family income demonstrated a significant negative causal effect. After adjusting for anxiety, depression retained a significant positive causal effect on CCS. These findings were consistent with those of previous studies. For instance, Xu^[7]^ conducted a systematic review and reevaluated the evidence to reveal the associations between depression, anxiety, and CCS incidence. Using MR analysis, they identified depression (β = 0.137, 95% CI: .035–.239, *P* = .008) as an independent risk factor for CCS. Fang^[16]^ demonstrated via MR analysis that depressive mood (*P* *<* .000) is genetically linked to CCS. Similarly, Lu ^[17]^ found that a 2.72-fold increase in genetically determined depression risk corresponded to a 14% increase in CAD and MI incidence, with smoking contributing 30.5% of the mediating effect. Qianjie et al^[18]^ reported results consistent with these findings. Gan^[19]^ retrospectively analyzed the medical records of patients undergoing coronary angiography using a decision tree classification model and identified significant differences in somatization symptom self-rating scale scores between coronary heart disease and noncoronary heart disease groups (*P* < .01). Incorporating the influence of negative emotions into medical decision-making systems enhances the diagnostic accuracy, which is consistent with the findings of this study. A recent genetic-based study^[20]^ suggested that family income and socioeconomic status (SES) had causal relationships with cardiovascular biomarkers and CAD risk (OR = 0.63, 95% CI: 0.49–0.79, *P* *=* .0001). Individuals with higher SES exhibit healthier behaviors such as regular exercise, low-salt diets, and nonsmoking habits. Therefore, increased economic income may alleviate the burden of cardiovascular diseases (CVD). Davari et al^[21]^ and other related studies^[12]^ support these findings, emphasizing the role of SES as a predictor of CVD risk factors and outcomes.

### 4.2. Two-step MR analysis

The 2-step MR analysis revealed 3 main trends:

1. Anxiety positively contributed to CCS through LDL levels (35.86%) and smoking initiation (10.26%). These findings align with data from relevant studies. For example, Zhen^[22]^ observed a significant positive correlation between anxiety scores, LDL-C levels, and smoking behavior among patients with CCS (*P* *<* .05). Pan^[23]^ reported a positive correlation between anxiety (depression) scores and LDL-C levels, with higher scores associated with more pronounced lipid abnormalities. LDL transports cholesterol into peripheral tissue cells, and elevated LDL levels promote cholesterol deposition in the vessel walls, leading to atherosclerotic plaque formation and exacerbating CCS progression. Smoking is a well-established risk factor for cardiovascular diseases, with harmful substances such as nicotine and tar damaging vascular endothelial cells, disrupting vascular integrity, promoting platelet aggregation and thrombosis, causing vasoconstriction and hypertension, and increasing the risk of CCS. These conclusions are consistent with the findings of this study.
2. Depression exerts bidirectional effects on CCS through 2 immune pathways, IL-10 (negative causal effect, 34.20%) and TRAIL (positive causal effect, 7.38%). Clinical studies have confirmed the presence of significant abnormalities in the cytokine profiles of patients with depression. Zheng et al^[24]^ compared serum samples from 31 patients with depression and 25 healthy controls and found elevated IFN-γ and IL-10 levels in individuals with depression (*P* < .01), indicating immune dysregulation. IL-10, a core anti-inflammatory cytokine secreted by Th2 cells, monocytes, and macrophages, inhibits pro-inflammatory factors such as TNF-α and IL-6, reducing vascular endothelial inflammation and injury, while suppressing atherosclerotic plaque progression.^[25]^ The compensatory increase in IL-10 among depressed patients may represent a self-protective response to chronic inflammation, delaying plaque formation or rupture and exerting a protective effect on CCS (34.20%). This aligns with IL-10’s “anti-inflammatory-repair” function, suggesting its potential as a therapeutic target for depression-related cardiovascular protection. Conversely, TRAIL expression is regulated by IFN-γ, which is primarily secreted by the activated T and NK cells. In depressive states, elevated IFN-γ upregulates TRAIL receptors on vascular endothelial, smooth muscle, and macrophage cell surfaces, inducing apoptosis and increasing plaque instability and rupture risk. Despite accounting for only 7.38% of the total effect, the pro-apoptotic pathway of TRAIL directly damages cardiovascular cells, potentially amplifying plaque vulnerability in patients with depression and those with chronic inflammation.
3. Household income negatively affected CCS through smoking initiation (12.83%), LDL (20.99%), and TGs (20.64%). Low income individuals face greater economic pressures, lower health awareness, and increased susceptibility to smoking, resulting in higher smoking rates. They also tend to consume unbalanced diets rich in low-cost, high-sugar, and high-fat foods, have reduced physical activity levels, and experience difficulties accessing timely medical care. These factors contribute to elevated LDL and TG levels, and increase the risk of CCS. The findings of Davari etal^[21]^ and other related study^[26]^support these conclusions.

## 5. Conclusions

Anxiety, depression, and diarrhea significantly increased the risk of CCS, whereas higher household income reduced this risk. Anxiety, depression, and household income indirectly influence CCS through their effects on smoking behavior, inflammatory responses, and blood lipid profiles. These findings provide actionable clinical guidance – integrating mental health screening, gastrointestinal symptom management, economic support, and targeted interventions for smoking, inflammation, and blood lipids into CCS prevention – and highlight the need for future longitudinal studies to confirm causal links and interventional trials to validate tailored strategies for reducing CCS incidence.

## Acknowledgments

The authors extend their sincere gratitude to the researchers and participants from the University of Bristol, the Institute of Epidemiology and Healthcare (IEU), UK Biobank, the FinnGen Consortium, and the EMBL-EBI/NHGRI-EBI GWAS Catalog for providing the essential data that supported the Mendelian randomization analysis conducted in this study. The authors also would like to thank Editage (www.editage.com) for English language editing.

## Author contributions

**Conceptualization:** Xiaotong Li, Yan Wang.

**Data curation:** Xiaotong Li.

**Formal analysis:** Xiaojing Guo.

**Methodology:** Xiaotong Li, Xiaojing Guo.

**Software:** Xiaotong Li.

**Supervision:** Yan Wang.

**Validation:** Chunhua Song.

**Visualization:** Xiaotong Li.

**Writing – original draft:** Xiaotong Li.

**Writing – review & editing:** Xiaotong Li, Xiaojing Guo.

## Supplementary Material


