# Supplementary material for: Exploring the causal pathways and mediating effects of sociopsychological factors on chronic coronary syndrome: A Mendelian randomization study
Source: Medicine (Baltimore). 2025 Nov 28;104(48):e45971. doi: 10.1097/MD.0000000000045971 (PMC12662325; doi:10.1097/MD.0000000000045971)

Supplementary materials

Table1. Genetic variation information of social psychological factors

Exposure	SNP	EA	NEA	beta	SE	*p*
Anxiety	rs10034259	C	A	-0.03284	0.005509	2.52E-09
	rs10496746	C	T	0.027104	0.004813	1.78E-08
	rs1077393	G	A	0.032723	0.004374	7.35E-14
	rs10831412	A	T	-0.0276	0.004428	4.54E-10
	rs10896636	G	C	0.026815	0.004642	7.65E-09
	rs10959797	A	G	-0.03383	0.005328	2.16E-10
	rs1109344	G	A	-0.02468	0.00443	2.51E-08
	rs11191419	A	T	-0.03548	0.004658	2.57E-14
	rs11237488	T	C	-0.03714	0.0067	2.97E-08
	rs113173628	T	C	0.035561	0.005403	4.65E-11
	rs11682716	T	G	0.027264	0.004452	9.15E-10
	rs12102776	T	C	-0.02677	0.004682	1.09E-08
	rs12365385	C	T	-0.02886	0.004554	2.36E-10
	rs13213752	T	C	-0.03057	0.0044	3.71E-12
	rs13262595	A	G	0.025308	0.004411	9.59E-09
	rs13289812	C	T	-0.03032	0.005378	1.72E-08
	rs138339382	C	T	-0.14581	0.026338	3.09E-08
	rs147321641	A	G	0.060996	0.010763	1.45E-08
	rs1660235	G	C	0.02975	0.004554	6.47E-11
	rs167915	T	A	-0.02942	0.004595	1.54E-10
	rs1833718	T	C	-0.03007	0.004572	4.79E-11
	rs187580	G	T	-0.03288	0.005158	1.83E-10
	rs194179	G	A	-0.02464	0.00444	2.85E-08
	rs2071754	C	T	0.033847	0.005451	5.33E-10
	rs226060	G	A	0.026685	0.004888	4.77E-08
	rs2282040	G	A	0.042677	0.007495	1.24E-08
	rs2367724	C	T	-0.02945	0.004665	2.75E-10
	rs236900	A	G	-0.03114	0.005681	4.22E-08
	rs2395183	C	T	-0.03044	0.00555	4.15E-08
	rs274632	A	C	-0.02832	0.00442	1.48E-10
	rs2759663	G	C	-0.04054	0.00527	1.44E-14
	rs2774012	C	T	-0.03212	0.005485	4.77E-09
	rs2881755	T	C	0.024573	0.004492	4.50E-08
	rs294379	T	A	-0.03067	0.004907	4.12E-10
	rs325451	C	T	-0.02993	0.004448	1.71E-11
	rs34636488	A	C	-0.02707	0.004494	1.71E-09
	rs4387544	T	C	-0.02568	0.004404	5.52E-09
	rs4444227	T	C	-0.03143	0.005284	2.71E-09
	rs55997507	G	C	-0.02947	0.004557	1.00E-10
	rs56322003	C	T	0.027223	0.004689	6.39E-09

	rs62250713	A	G	-0.04238	0.004554	1.33E-20
	rs62433356	A	G	-0.03337	0.006118	4.93E-08
	rs6439649	G	T	-0.0317	0.004462	1.22E-12
	rs6590229	T	C	0.026591	0.004839	3.92E-08
	rs6685497	A	T	0.026394	0.004451	3.02E-09
	rs7043325	A	G	0.02833	0.00444	1.77E-10
	rs7147721	G	A	0.031072	0.004411	1.87E-12
	rs7421968	G	C	-0.02914	0.00502	6.49E-09
	rs7567451	G	T	-0.028	0.004954	1.57E-08
	rs908730	G	A	-0.02555	0.004467	1.06E-08
	rs922140	A	G	-0.02621	0.004474	4.69E-09
	rs9611522	T	C	0.029109	0.005095	1.11E-08
	rs9811585	G	T	0.031349	0.004513	3.75E-12
	rs9835772	T	A	-0.02899	0.00511	1.40E-08
	rs9964724	C	T	0.030014	0.004718	1.99E-10
MDD	rs10233018	A	G	-0.018	0.004	1.65E-07
	rs11587416	T	C	-0.026	0.006	3.46E-06
	rs11636582	T	G	-0.042	0.009	6.98E-07
	rs139560451	T	C	-0.059	0.013	2.88E-06
	rs148701159	A	G	0.035	0.008	3.74E-06
	rs1690818	T	C	-0.019	0.004	4.05E-07
	rs1877075	A	G	-0.022	0.005	2.00E-06
	rs1961982	A	G	-0.022	0.005	2.25E-06
	rs2017122	T	C	0.033	0.007	2.32E-06
	rs3783005	A	T	0.021	0.004	1.33E-06
	rs4810896	A	C	-0.017	0.003	9.00E-07
	rs4942916	T	C	-0.022	0.005	9.97E-07
	rs59659806	T	C	-0.022	0.004	6.01E-07
	rs61906135	A	C	-0.057	0.012	2.60E-06
	rs62100776	A	T	-0.025	0.004	8.45E-09
	rs6458013	T	C	0.023	0.005	1.02E-06
	rs652714	A	G	-0.023	0.005	8.95E-07
	rs6992714	T	C	0.02	0.004	9.32E-08
	rs7074335	T	C	0.035	0.007	4.57E-07
	rs72939513	A	G	-0.044	0.009	3.14E-06
	rs7300143	A	G	0.023	0.005	2.34E-06
	rs77508728	T	C	0.055	0.012	4.91E-06
	rs782212	T	C	-0.018	0.004	1.23E-06
	rs7973260	A	G	0.031	0.005	1.78E-09
	rs853679	A	C	-0.023	0.005	6.62E-07
	rs9427622	T	C	-0.021	0.004	4.75E-07
	rs9845113	C	G	0.018	0.004	4.64E-07
Constipation	rs10139484	T	G	-0.0526	0.0114	4.35E-06

	rs10223052	G	A	0.0546	0.0119	4.85E-06
	rs11648475	G	A	0.0546	0.0114	1.75E-06
	rs12934865	T	C	0.0558	0.0115	1.20E-06
	rs1376767	C	A	-0.0706	0.014	4.72E-07
	rs139180659	C	T	0.6488	0.1333	1.13E-06
	rs139714059	T	A	0.0672	0.0146	4.10E-06
	rs149635700	C	T	-0.1654	0.036	4.29E-06
	rs186529831	T	C	-0.2466	0.0512	1.50E-06
	rs2354178	A	G	-0.0592	0.0128	4.01E-06
	rs4083752	A	G	0.0565	0.0118	1.70E-06
	rs55694472	T	C	0.1641	0.0286	9.91E-09
	rs56029819	C	T	0.0757	0.0162	2.83E-06
	rs5757641	G	A	-0.0585	0.0121	1.29E-06
	rs59674167	T	C	-0.0718	0.0148	1.17E-06
	rs616812	C	T	-0.0631	0.0135	3.16E-06
	rs74287578	C	A	-0.1576	0.0323	1.05E-06
	rs75271724	G	T	0.2218	0.0456	1.14E-06
	rs79086497	A	G	-0.0811	0.0176	3.93E-06
Diarrhea	rs12644715	C	T	0.421712	0.0916148	4.16e-06
	rs13346414	A	G	0.667159	0.142128	2.67838e-06
	rs140052237	C	T	1.43191	0.279834	3.10455e-07
	rs147579663	G	A	2.15286	0.457849	2.57485e-06
	rs172962	A	G	-0.355736	0.0754265	2.40143e-06
	rs2523466	T	G	0.427886	0.0896942	1.83772e-06
	rs33073	A	T	-0.379752	0.0773655	9.17519e-07
	rs3947061	A	T	0.441603	0.0947975	3.18713e-06
	rs4713966	A	T	0.380053	0.0800269	2.0436e-06
	rs73071385	G	C	0.578101	0.115587	5.69016e-07
	rs78469462	T	C	0.678611	0.144963	2.85118e-06
	rs942679	A	C	0.367645	0.0748409	8.99921e-07
Nausea and vomiting	rs10806772	G	A	0.000704725	0.000154178	4.9000e-06
	rs11108960	C	G	0.000800114	0.000162385	8.3000e-07
	rs2131737	C	G	-0.000700452	0.000153134	4.7999e-06
	rs6909095	A	C	0.000749125	0.000158981	2.4999e-06
	rs7533146	C	T	0.000742386	0.000161852	4.4999e-06
Type A behavior	rs1010235	A	T	0.2928	0.0617	2.192e-06
	rs137925278	A	C	-0.6177	0.1284	1.58e-06
	rs56160063	G	A	-0.1334	0.0278	1.677e-06
	rs58993351	C	T	0.4162	0.0901	4.014e-06
	rs6084912	C	A	0.1878	0.036	1.891e-07
	rs6122429	C	T	-0.1559	0.0339	4.369e-06

	rs9952361	G	A	0.169	0.0367	4.223e-06
Social deprivation	rs12042107	C	T	-0.0380952	0.00590783	1.13E-10
	rs12150771	G	A	-0.0397322	0.00716227	2.90E-08
	rs6931604	T	C	-0.0335421	0.00599885	2.25E-08
	rs6968125	T	C	-0.0333109	0.00589691	1.61E-08
	rs704067	A	G	0.0345368	0.0059107	5.12E-09
	rs8614	A	C	0.0465794	0.00759762	8.74E-10
	rs989532	G	A	0.0346081	0.00616128	1.94E-08
Household income	rs10429582	C	T	0.01713	0.002	1.98E-17
	rs11588857	A	G	0.01154	0.002	9.99E-09
	rs11943536	T	C	0.01112	0.002	3.92E-08
	rs12119149	T	C	0.01265	0.002	5.24E-10
	rs12151248	T	C	-0.01242	0.002	4.04E-10
	rs12614880	G	A	0.01461	0.002	4.66E-13
	rs1263412	G	A	0.01143	0.002	1.40E-08
	rs12954483	G	A	-0.0126	0.002	5.80E-10
	rs12967855	G	A	0.01234	0.002	2.01E-09
	rs139897186	G	C	-0.0112	0.002	1.71E-08
	rs1455350	A	T	-0.01152	0.002	1.16E-08
	rs151114485	A	G	-0.01141	0.002	5.96E-09
	rs159365	G	A	0.01566	0.002	9.04E-15
	rs17652520	A	G	-0.01385	0.002	4.56E-12
	rs1906252	A	C	0.01728	0.002	1.39E-17
	rs2332719	G	A	-0.01333	0.002	1.16E-10
	rs2563332	T	C	-0.01156	0.002	9.90E-09
	rs28558559	C	T	0.01122	0.002	4.11E-08
	rs28896784	G	T	0.01123	0.002	3.60E-08
	rs306755	C	T	0.01192	0.002	3.82E-09
	rs32940	C	T	0.01107	0.002	3.73E-08
	rs37976	T	C	0.01198	0.002	3.14E-09
	rs4727618	A	G	-0.01145	0.002	1.67E-08
	rs537160	G	A	-0.01286	0.002	2.34E-10
	rs6477496	T	C	0.01126	0.002	2.85E-08
	rs6699397	G	A	-0.0122	0.002	1.59E-09
	rs7597007	T	G	-0.01221	0.002	1.46E-09
	rs9517310	T	C	0.01112	0.002	3.09E-08
	rs9822268	A	G	0.01316	0.002	6.98E-11

Supplementary materials

Figure 1.

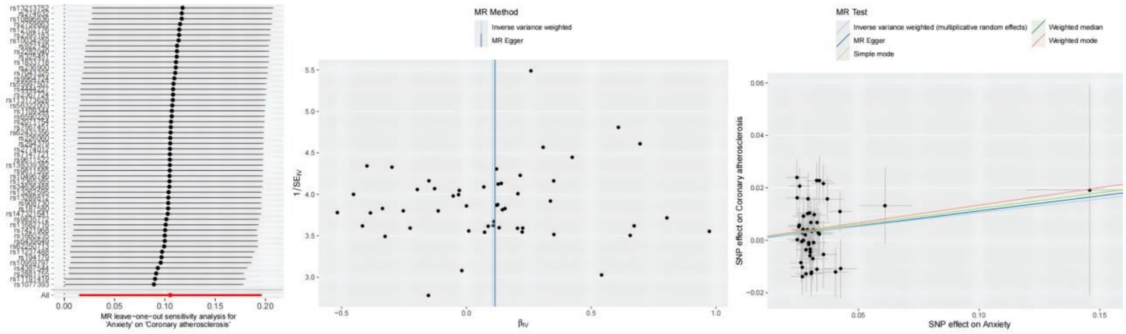

Supplementary materials

Figure 2.

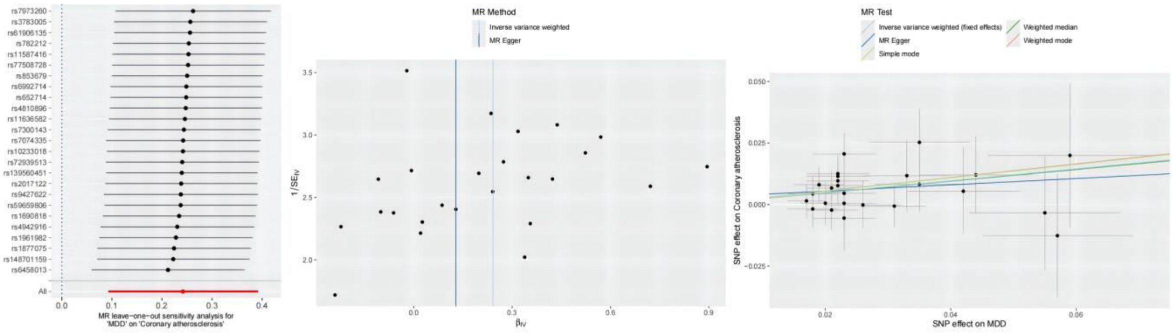

Supplementary materials

Figure 3.

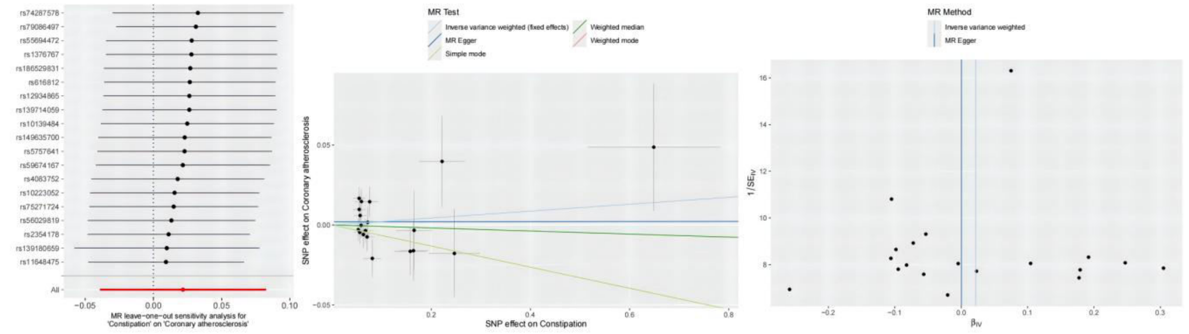

Supplementary materials

Figure 4.

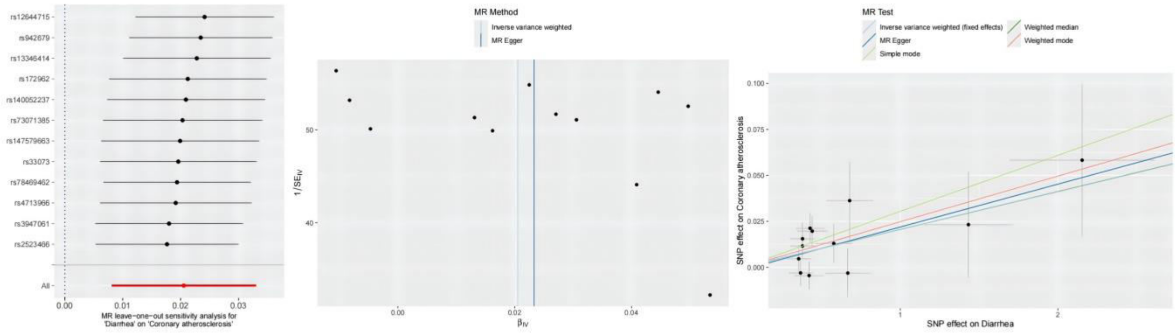

Supplementary materials

Figure 5.

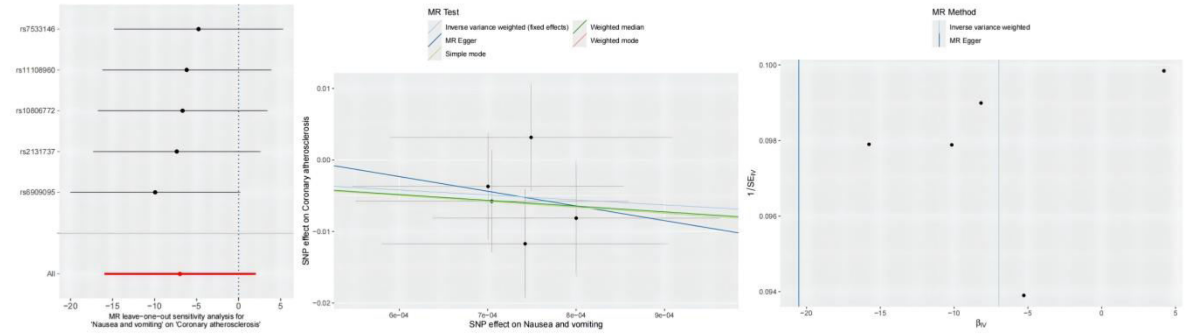

Supplementary materials

Figure 6.

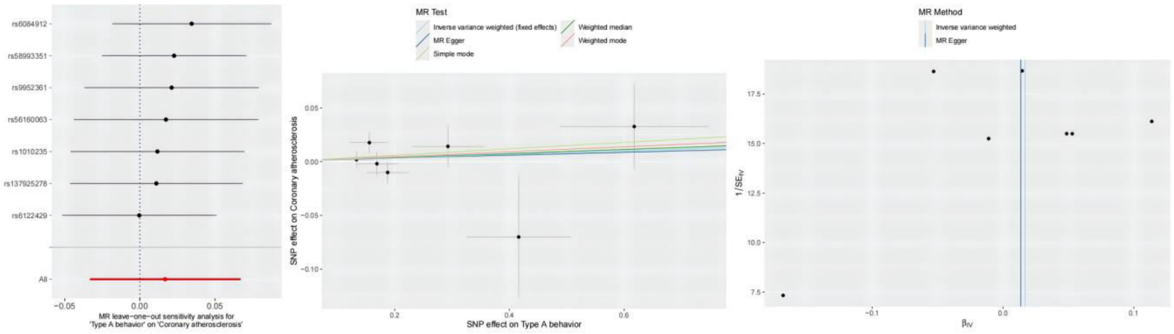

Supplementary materials

Figure 7.

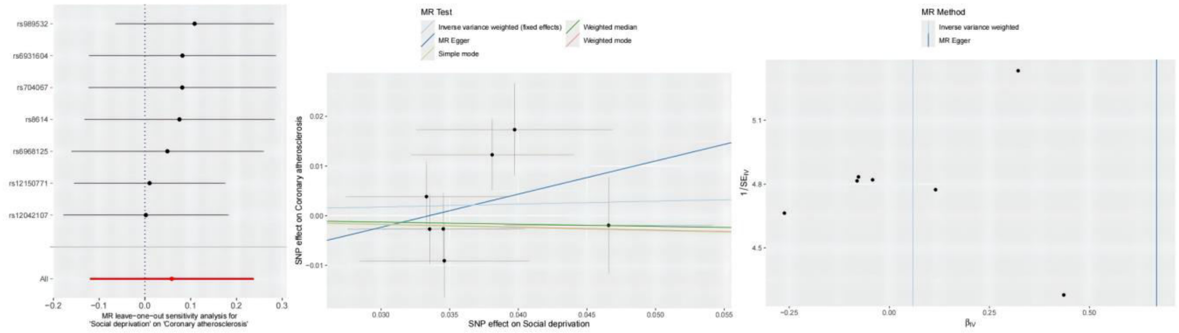

Supplementary materials

Figure 8.

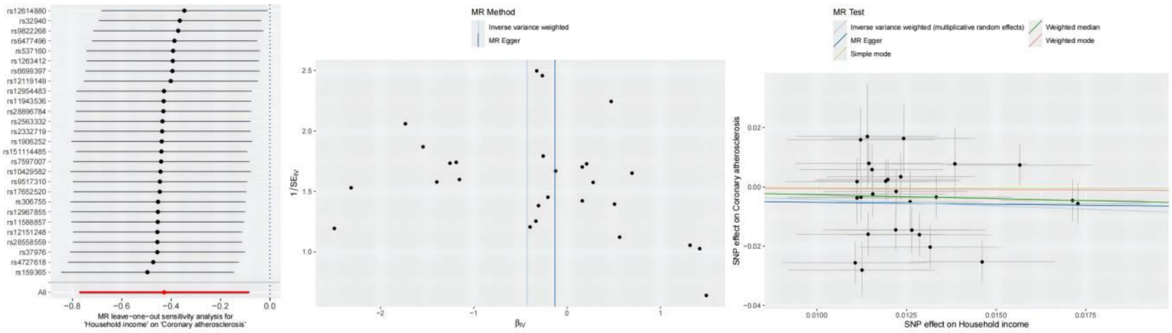

Supplementary materials

Figure 9.

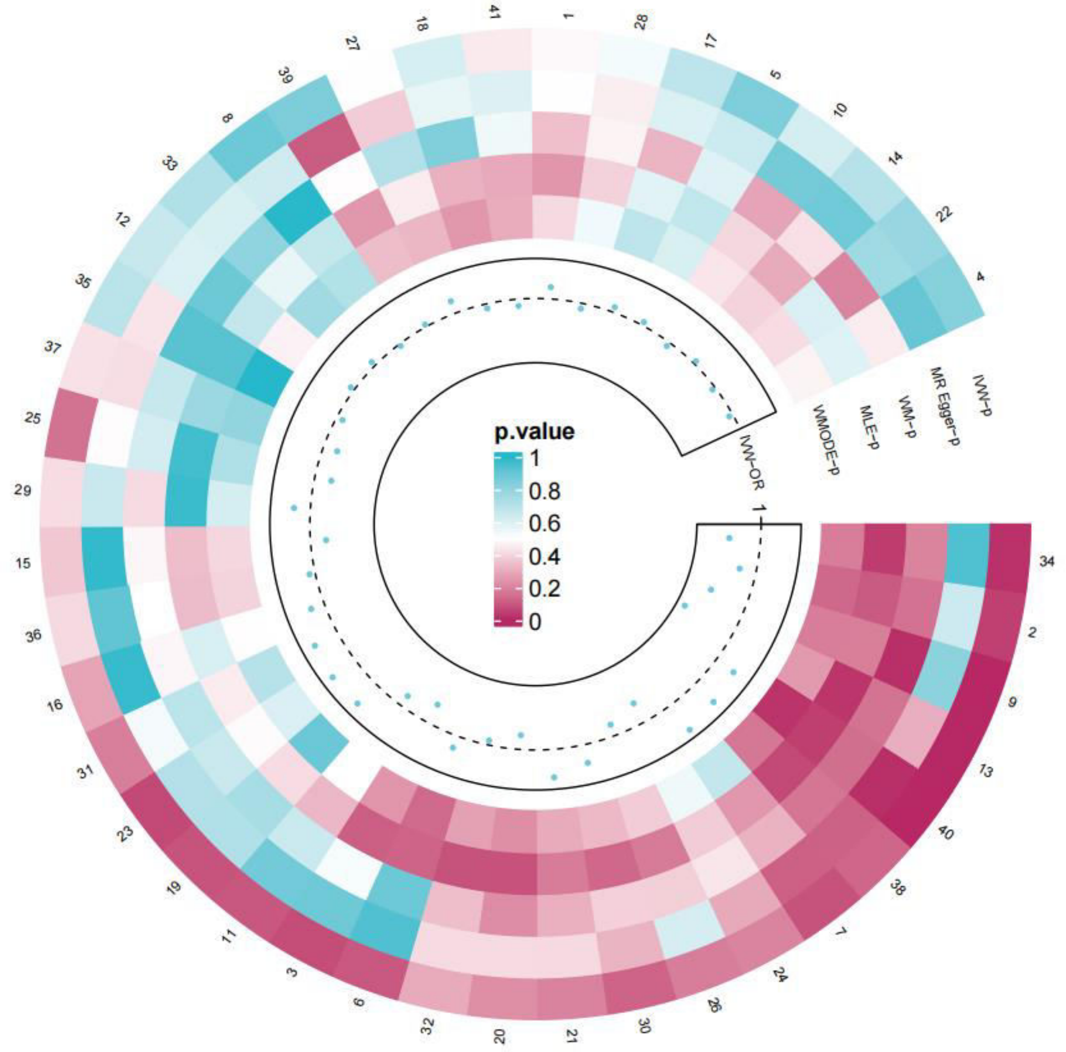

Supplementary materials

Figure 10.

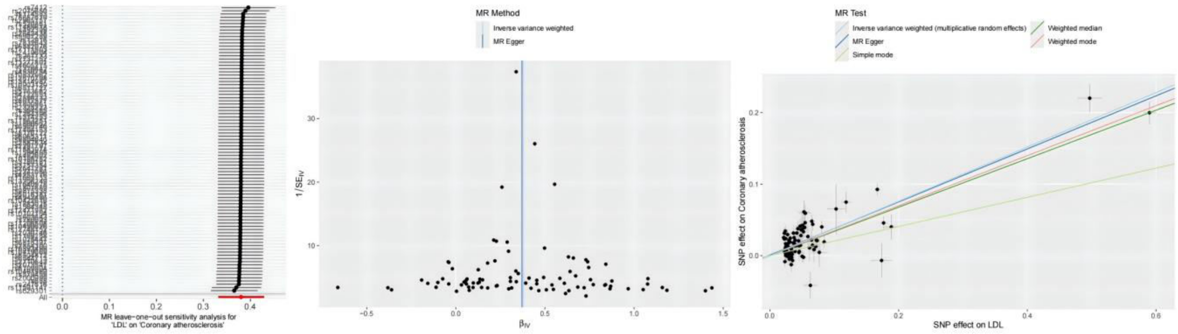

Supplementary materials

Figure 11.

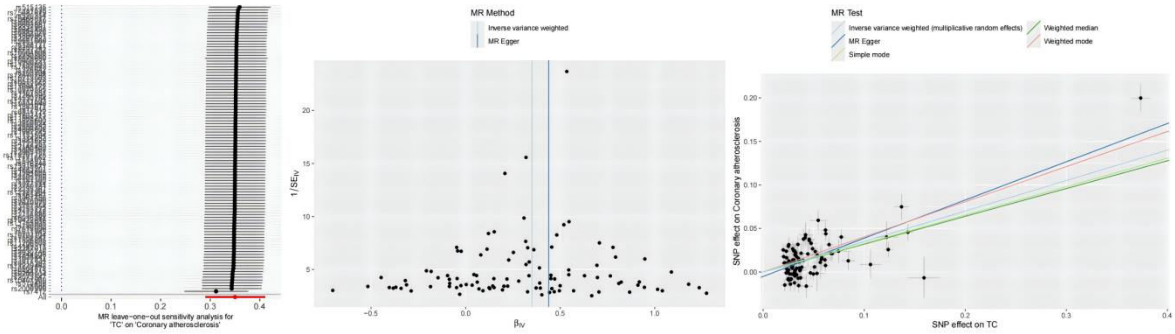

Supplementary materials

Figure 12.

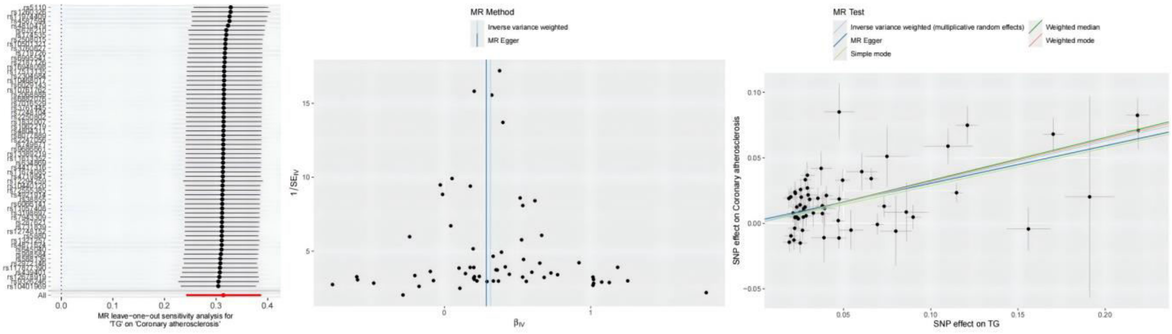

Supplementary materials

Figure 13.

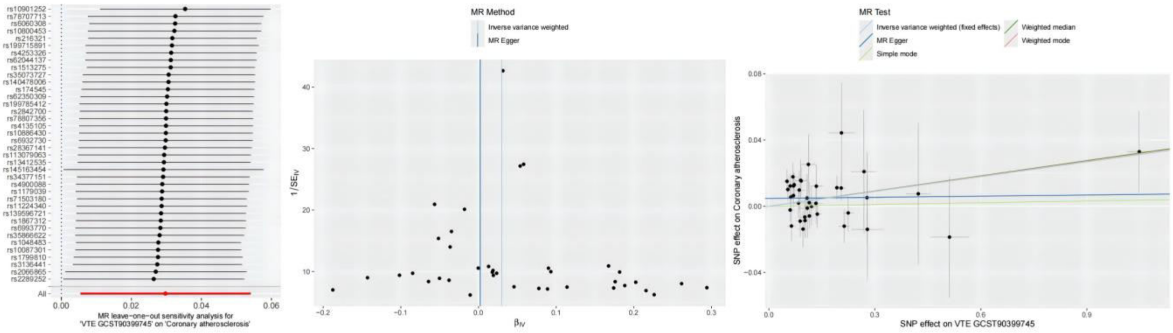

Supplementary materials

Figure 14.

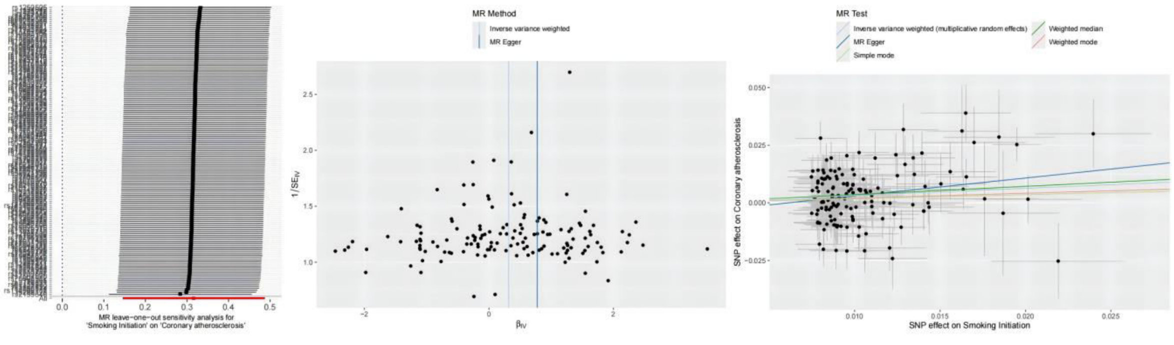

Supplementary materials

Figure 15.

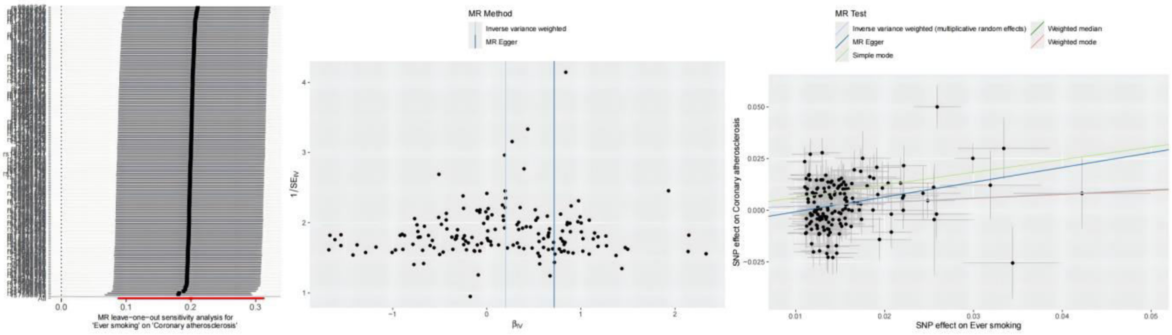

Supplement: Supplementary file 1 [file medi-104-e45971-s001.pdf]
